# A Rare Case of Congenital Rhabdomyosarcoma with Review of the Literature

**DOI:** 10.1155/2013/518952

**Published:** 2013-11-20

**Authors:** Gautam Bir Singh, Rubeena Arora, Deepak Kumar, Manjula Jain, Vandana Puri

**Affiliations:** ^1^Department of Otorhinolaryngology & Head-Neck Surgery, Lady Hardinge Medical College & Associated Hospital, Shaheed Bhagat Singh Marg, New Delhi 110029, India; ^2^Department of Pathology, Lady Hardinge Medical College & Associated Hospital, Shaheed Bhagat Singh Marg, New Delhi 110029, India

## Abstract

We present a rare case of rhabdomyosarcoma of lip in a neonate with multiple lesions within the head and necksub site hitherto unreported in the medical literature. This case report also reviews the scant medical literature on neonatal rhabdomyosarcoma.

## 1. Introduction

Rhabdomyosarcoma (RMS) is a malignant soft tissue neoplasm of the skeletal muscle origin. The tumour accounts for 4–8% of all malignancies in children of less than 15 years of age [1]. It has a bimodal peak incidence, the first occurring at the age of 2–6 years and the other occurring at adolescence [2]. RMS also has a strong male preponderance. Though congenital RMS has been reported in the medical literature, it is extremely rare [1, 3]. With this background, we present a case of neonatal rhabdomyosarcoma with unusual clinical presentation.

## 2. Case Report

An 11-day-old neonate presented to the “Paediatric Otorhinolaryngology OPD” of our Kalawati Saran Paediatric Hospital, attached to Lady Hardinge Medical College, New Delhi, India, a tertiary care university teaching hospital with a swelling in the upper lip since birth and complaint of difficulty in suckling. In general the baby was irritable and always crying.

**Figure 1:**
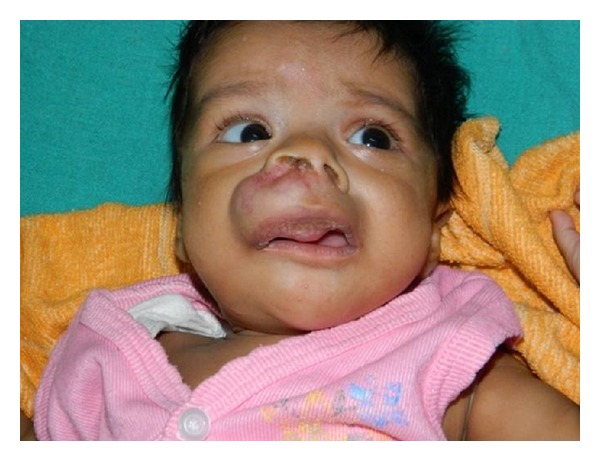
Clinical photograph of the patient with lesion in upper lip.

A large ovoid swelling was seen in the upper lip and adjoining part of upper face. The lesion involved the anterior nares causing stenosis. On the buccal surface, the skin over the swelling was normal with no sinus, punctum, or ulceration. The alveolus was free of the swelling. The swelling measured 5 × 3 cm in maximum dimensions. The swelling was tender, firm in consistency and the skin over it was indurated in patches with engorgement of veins (Figure 1). A differential diagnosis of odontogenic tumour, dermoid, solitary fibromatosis, gingival granular cell tumour, rhabdomyosarcoma and neurofibroma was considered.

**Figure 2:**
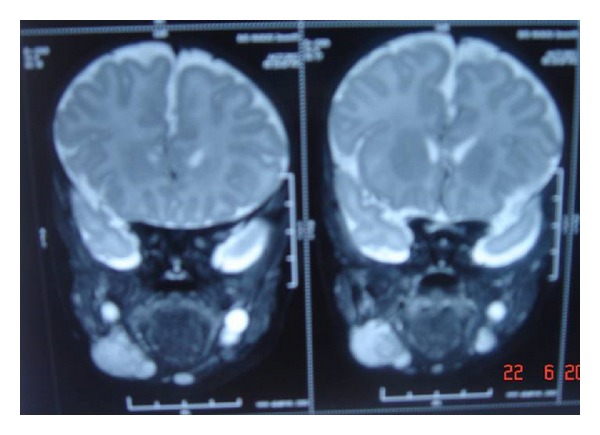
MRI scan showing lesion in parapharyngeal space.

**Figure 3:**
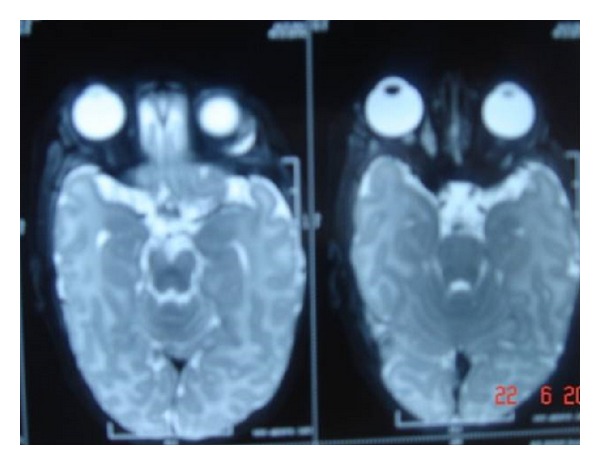
MRI scan showing lesion in the orbit.

**Figure 4:**
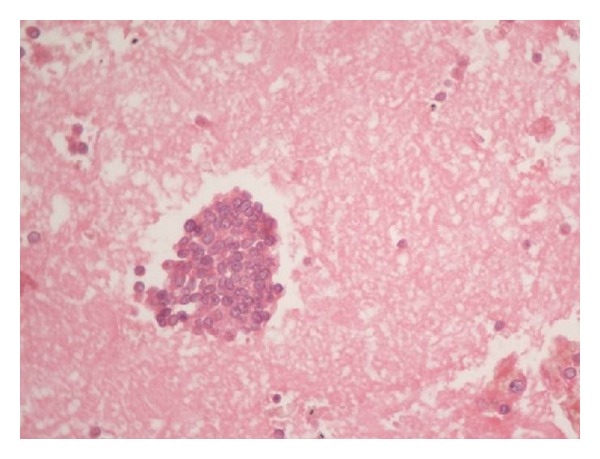
Cell block showing cluster of small round cells.

**Figure 5:**
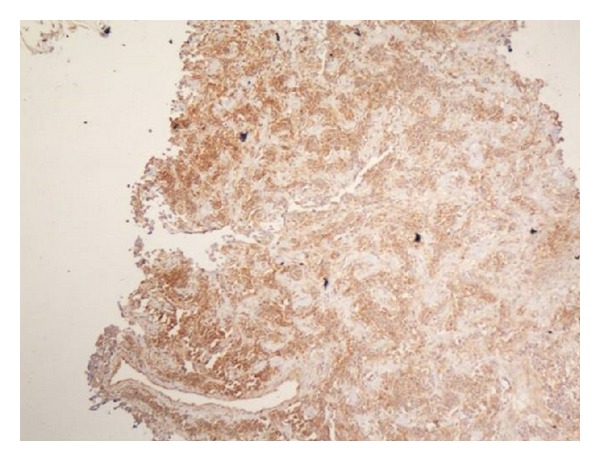
Immunocytochemistry: leucocyte common antigen positive for Desmin.

**Figure 6:**
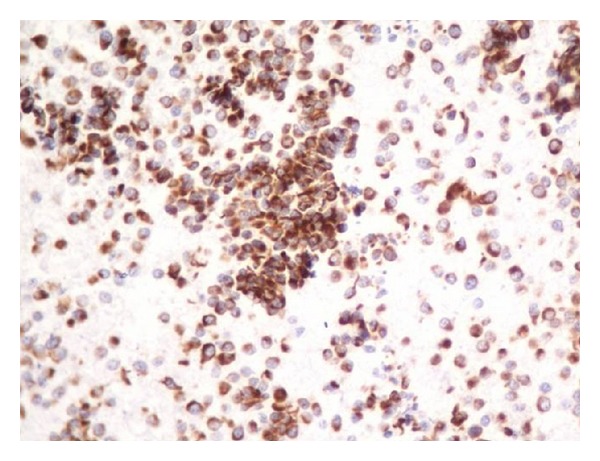
Immunocytochemistry: leucocyte common antigen positive for Myogenin.

Patient underwent CT scan and MRI scan which revealed soft tissue intensity lesions in the lip, orbit and submandibular region (parapharyngeal space) (Figures 2 and 3). Fine needle aspiration and biopsy from the lip lesion revealed population of round cells which were arranged in clusters as well as dispersed cell population. These cells were small with scant-to-moderate amount of cytoplasm, round-to-oval nucleus, fine chromatin, and inconspicuous nucleolus. No cytoplasmic vacuoles or rosettes were seen (Figure 4). Immunocytochemistry was put up for LCA, CD99, Desmin and Myogenin to confirm the diagnosis. Leucocyte common antigen (LCA) was strongly positive for Desmin and Myogenin (Figures 5 and 6). On the basis of histopathology, though initially a differential diagnosis of lymphoma and Ewing's sarcoma was also considered, immunocytochemistry clinched the diagnosis of embryonal rhabdomyosarcoma.

In view of multiple lesions, the said patient underwent chemotherapy with Vincristine, Actinomycin, Cyclophosphamide, and Dexamethasone. Subsequently, patient developed metastatic lesions in bone and lungs. Unfortunately, despite aggressive therapy, patient succumbed to his disseminated disease 3 months later.

## 3. Discussion

RMS is uncommon in neonates; thus, not much medical literature is available on the cited subject. Etiology of RMS is obscure. However, it is associated with genetic conditions like: neurofibromatosis and Li-Fraumeni syndrome [2, 4]. The commonest sites of involvement of RMS are head and neck, genitourinary tract, retroperitoneum, and extremities [5]. Within the head and neck region, RMS is divided into three types on the basis of anatomical distribution [1]: orbital, parameningeal, and nonorbital-nonparameningeal. The parameningeal type involves pterygopalatine and infratemporal fossa, paranasal sinuses, and middle ear. These are associated with poor prognosis. The nonorbital-nonparameningeal involves scalp, face, parotid, and oral cavity. These are associated with good prognosis. It would be prudent to note that RMS of the oral cavity is rare and is seen in only 10% of all diagnosed cases, tongue being the commonest site [6].

Histopathologically, the tumour is classified as embryonic, alveolar, and pleomorphic types [2]. The embryonal is the commonest and is further divided into botryoid and spindle cell types. The alveolar has the worst prognosis. The high population count of small round cells makes it imperative to keep Ewing's sarcoma and lymphoma as other important histopathological differentials. The confirmation of diagnosis is made by immunocytochemistry using a panel of markers: Desmin, Myogenin, CC99, and CCA. Alveolar type can also be characterized by specific genetic changes [3, 7]. The translocations involve two PAX genes PAX3 and PAX7 (on chromosome 2 and 1, resp.). These translocations result in the fusion genes between PAX 3 and PAX7 and the transactivation domain of the FKHR gene on chromosome 13. These resultant fusion genes are PAX3-FKHR and PAX7-FKHR. These translocations result in an alteration of biological activity at the protein level and are thought to influence tumourigenic behavior by impacting the control of tumour cell growth, apoptosis, differentiation, and motility. Both fusion genes provide a unique diagnostic marker for alveolar rhabdomyosarcoma. Metastasis of RMS is by hematogenous, direct and lymphatic routes [1].

These tumours have no specific clinical presentation [1]. Orofacial rhabdomyosarcoma may present as a painless cutaneous nodule or a rapidly growing facial mass, as was seen in this case. They may be associated with paresthesia, pain, and facial nerve palsy. In absence of characteristic diagnostic features, they are frequently misdiagnosed. Diagnosis is thus made by the aid of investigations like [2, 4]: fine needle aspiration, CT-scan, and MRI scan (regarded as the best radiological investigation) and finally confirmed by biopsy. In the case of head and neck lesions: lumber puncture with cytological examination, skeletal survey, bone scan, and bone marrow biopsy is necessary for a complete systemic evaluation to rule out any metastasis.

Multidisciplinary approach in the management of RMS with surgery, radiotherapy, and chemotherapy has dramatically improved the prognosis for this potentially fatal tumour, with the current medical literature reporting a five-year survival rate of 74–77% [1, 2, 6, 8].

This case merits discussion on many accounts. This case is reported because of extreme rarity of RMS to occur in neonates and in the oral cavity too (upper lip). Moreover, the lesion within a short span of 10 days increased rapidly in size causing symptoms in the neonate. Further, the radiological examination revealed multiple subsite involvement within the head and neck region. Though it has been noted in congenital RMS that the disease may be metastatic at the time of birth [9], in a massive Internet/PubMed search authors could find no case of multiple metastatic lesions within the same subsite. This raises a medical dilemma: are these multiple primaries? If not, then which is the true primary? The subject is open to debate. However, in this context it would be pertinent to note that, within the head and neck region, the orbit is the commonest site of involvement for RMS. And interestingly, no CNS metastasis was recorded in this case. Last but not least, this case also highlights the poor prognosis associated with RMS especially in neonates, although there is no clear evidence that biology of RMS is different in infants. The review of English medical literature on neonatal RMS cites the following factors for this poor prognosis [6, 8, 10]:

1. age less than 1 year;
2. delayed diagnosis;
3. less aggressive treatment attributed to the side effects of the chemo-radiation therapy;
4. metastatsis;
5. genetically: PAX3-FHR translocations.

This unusual presentation of rhabdomyosarcoma of the lip in a neonate prompted us to share our clinical experience with other members of medical fraternity.
